# Machine Learning Model Comparison in the Screening of Cholangiocarcinoma Using Plasma Bile Acids Profiles

**DOI:** 10.3390/diagnostics10080551

**Published:** 2020-08-02

**Authors:** Davide Negrini, Patrick Zecchin, Andrea Ruzzenente, Fabio Bagante, Simone De Nitto, Matteo Gelati, Gian Luca Salvagno, Elisa Danese, Giuseppe Lippi

**Affiliations:** 1 Department of Laboratory Medicine, University-Hospital of Padova, 35128 Padova, Italy; davidenegrini@outlook.com; 2 Clinical Biochemistry Section, Department of Neurological, Biomedical and Movement Sciences, University of Verona, 37134 Verona, Italy; email@patrickzecchin.com (P.Z.); simone.denitto@studenti.univr.it (S.D.N.); matteo.gelati@univr.it (M.G.); gianluca.salvagno@univr.it (G.L.S.); giuseppe.lippi@univr.it (G.L.); 3 Department of Surgery, Division of General Surgery, Unit of Hepato-Pancreato-Biliary Surgery, University of Verona, 37134 Verona, Italy; andrea.ruzzenente@univr.it (A.R.); fabio.bagante@univr.it (F.B.)

**Keywords:** machine learning, artificial intelligence, bile acids, cholangiocarcinoma, screening

## Abstract

Bile acids (BAs) assessments are garnering increasing interest for their potential involvement in development and progression of cholangiocarcinoma (CCA). Since machine learning (ML) algorithms are increasingly used for exploring metabolomic profiles, we evaluated performance of some ML models for dissecting patients with CCA or benign biliary diseases according to their plasma BAs profiles. We used ultra-performance liquid chromatography tandem mass spectrometry (UHPLC-MS/MS) for assessing plasma BAs profile in 112 patients (70 CCA, 42 benign biliary diseases). Twelve normalisation procedures were applied, and performance of six ML algorithms were evaluated (logistic regression, k-nearest neighbors, naïve bayes, RBF SVM, random forest, extreme gradient boosting). Naïve bayes, using direct bilirubin concentration for normalisation of BAs, was the ML model displaying better performance in the holdout set, with an Area Under Curve (AUC) of 0.95, 0.79 sensitivity, 1.00 specificity. This model, also characterised by 1.00 positive predictive value and 0.73 negative predictive value, displayed a globally excellent accuracy (86.4%). The accuracy of the other five models was lower, and AUCs ranged 0.75–0.95. Preliminary results of this study show that application of ML to BAs profile analysis can provide a valuable contribution for characterising bile duct diseases and identifying patients with higher likelihood of having malignant pathologies.

## 1. Introduction

Machine learning (ML), a major branch of artificial intelligence (AI), is conventionally defined as a science where computer programs learn associations of predictive power from examples in data. Considering the large amount of digital information that medical devices and instruments can now generate, ML is increasingly applied to laboratory diagnostics for its ability to exploit predefined algorithms and statistical techniques for improving screening, identification, diagnosis, prognostication and therapeutic monitoring of human diseases [1].

Although some applications of neural networks have been used in healthcare and medical research already in the early nineties [2], the growing interest in AI and its related technologies has been recently confirmed by clearance for clinical use of some SaMD (software as medical device) and AI applications by the US Food and Drug Administration (FDA) [3,4].

ML is an extension of traditional statistical approaches, where a computer program learns to perform tasks or make decisions automatically, basing its decisions and actions on calculations on training data [5]. Considering this definition in a broader perspective, thus including all forms of data-driven approaches, the use of ML (especially in the form of deep learning algorithms) could also be extended for analysis of metabolomic profiles in blood and other biological fluids [3,6], with the specific aim of discovering some peculiar “fingerprints” characterising a vast array of human diseases [7].

Each ML algorithm has different advantages or disadvantages, which can be theoretically defined considering the data and how the classification task is performed. Such information cannot always be verified a priori, so that empirical comparison of classification performance remains necessary under some circumstances [8]. An accepted approach for evaluating the performance of different classification algorithms is to train, and successively test them, in a setting called “supervised learning” (when correct classification for all the dataset rows is available a priori). The diagnostic accuracy can then be assessed using ROC (receiver operating characteristic) curves, which provide precise performance measures such as the area under the curve (AUC) with its combined diagnostic sensitivity and specificity [7,9,10].

Cholangiocarcinoma (CCA) is the second most common primary hepatic tumour worldwide, with an incidence rate that has consistently increased over the past 30 years. The pathogenesis of CCA is complex and multi-factorial, mostly characterised by deregulation of various signalling networks. Despite several efforts that have been made in the past few years for garnering a better understanding of CCA biology, the intricate network of molecular mechanisms responsible for early and widespread dissemination of this malignancy remains mostly elusive. Nonetheless, the assessment of bile acids (BAs) has recently gained increasing interest for their potential involvement in development and progression of this type of cancer [11].

Next generation assays have been developed to allow their quantification in biological fluids; to date, techniques based on liquid chromatography tandem mass spectrometry (LC-MS/MS) are considered the gold standard for analysis of BAs profile in biological samples. These methods provide optimal resolution combined with enhanced diagnostic sensitivity and specificity [12,13].

Therefore, this study was aimed to evaluate the performance of some ML models for dissecting patients with CCA or benign biliary diseases according to their plasma BAs profiles.

## 2. Materials and Methods

A total number of 112 patients (69 males, 43 females; median age 70 years, range 20 to 87 years) were enrolled. All participants provided an informed consent for participation to this study, which was carried out in accordance with the Declaration of Helsinki and was approved by the local ethical committee (University Hospital of Verona; 24113CESC, May 16, 2017).

Plasma BAs quantification was performed using a validated ultra-performance liquid chromatography tandem mass spectrometry (UHPLC-MS/MS) technique, as extensively described elsewhere [14]. Briefly, separation and quantification of individual BAs was performed on a Acquity UPLC I-Class System FTN coupled with a Xevo TQ-S micro MS/MS (Waters Corporation, Milford, MA, USA) detector operating in multiple reaction monitoring (MRM) and electrospray negative ionisation mode (ESI-). Investigated BAs (*n* = 15) were the following: Tauroursodeoxycholic acid (TUDCA), taurocholic acid (TCA), glycoursodeoxycholic acid (GUDCA), glycocholic acid (GCA), taurochenodeoxycholic acid (TCDCA), taurodeoxycholic acid (TDCA), cholic acid (CA), ursodeoxycholic acid (UDCA), glycochenodeoxycholic acid (GCDCA), hyodeoxycholic acid (HDCA), glycodeoxycholic acid (GDCA), taurolithocholic acid (TLCA), chenodeoxycholic acid (CDCA), glycolithocholic acid (GLCA), deoxycholic acid (DCA); six internal standards were used: Taurocholic acid-d4 (d4-TCA), glycocholic acid-d4 (d4-GCA), cholic acid-d4 (d4-CA), ursodeoxycholic acid-d4 (d4-UDCA), chenodeoxycholic acid-d4 (d4-CDCA), deoxycholic acid-d4 (d4-DCA). An eight-point calibration curve was used starting from methanolic standards, with a linearity between 5 and 5000 ng/mL. Instrument data was collected and analysed using MassLynx V4.2 SCN977 (Waters Corporation, Milford, Massachusetts, United States).

BAs concentrations lower than the lower limit of quantitation (5 ng/mL for each bile acid) were imputed as 5/sqrt(2) ng/mL [15,16]. Total and direct bilirubin concentrations were determined on the same sample used for BAs profile, using a Roche Cobas 8000 clinical chemistry analyser (Roche Diagnostics, Risch-Rotkreuz, Switzerland) and using proprietary reagents.

According to the results of histological examination, patients were classified as having CCA (*n* = 70; including intrahepatic, perihilar and distal CCA) or benign biliary diseases (*n* = 42; including bile ducts stenosis, chronic cholecystitis, calculous cholecystitis).

**Figure 1:**
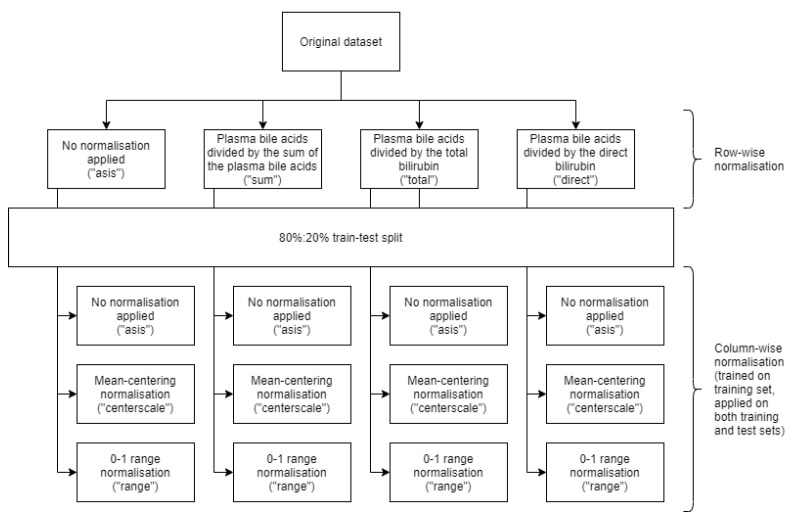
Normalisation processes flowchart applied to the dataset.

Statistical analyses were performed using R version 3.6.1 (build 2019-07-05) (R Foundation for Statistical Computing, Vienna, Austria) running on Ubuntu 19.10 64-bit (Canonical Group Limited, London, UK) operating system. The original dataset was composed of 21 different parameters (benign/malign, age in years, sex, total bilirubin, direct bilirubin, sum of plasma BAs and the 15 individual BAs), and underwent two different normalisation steps, for a total of 12 different processings, as shown in Figure 1. First, four normalisations were applied row-wise, in order to standardise with respect to different quantities. In particular, we chose to normalise plasma BAs concentrations dividing them by the sum of BAs, by total bilirubin concentration or by direct bilirubin concentration, or not normalising data row-wise. The dataset was then split in training and test (or holdout) sets, creating a 80:20% split (90 rows in training dataset, 22 rows in test/holdout set) stratified on the dependent variable (the benign/malign classification). Finally, for each of the four normalisations, three additional column-wise standardisations were tested. We applied mean-centring normalisation (that is, imposing the column values to be centred in 0 and to have a unitary variance by subtracting the mean and dividing by the standard deviation) and 0–1 range normalisation (having the values in the [0;1] interval by subtracting the minimum value and dividing by the value range), or not normalising data column-wise. The computation was performed on the training set and applied on both training and test set, to avoid information leakage.

The training dataset was further split, exploiting a 20-times repeated 5-fold cross-validation [17] to perform a hyperparameter tuning and optimise model performance. The hyperparameter selection was carried out with randomly chosen trials (up to 50 combinations) [18] and had as objective function the maximisation of the AUC metrics.

We evaluated six common classification algorithms: Logistic regression (R package “LiblineaR” [19]), k-nearest neighbors (R package “kknn” [20]), naïve bayes (R package “naivebayes” [21]), RBF support vector machines (R package “kernlab” [22]), random forest (R package “ranger” [23]) and extreme gradient boosting (R package “xgboost” [24]). For each ML algorithm applied to the dataset, analyses of AUC with its related diagnostic sensitivity and specificity were performed.

Visual inspection of datasets and output models were finally performed, based on the uniform manifold approximation and projection (UMAP) technique to plot data in a 2D space [25], using R package “umap” [26].

## 3. Results

**Figure 2:**
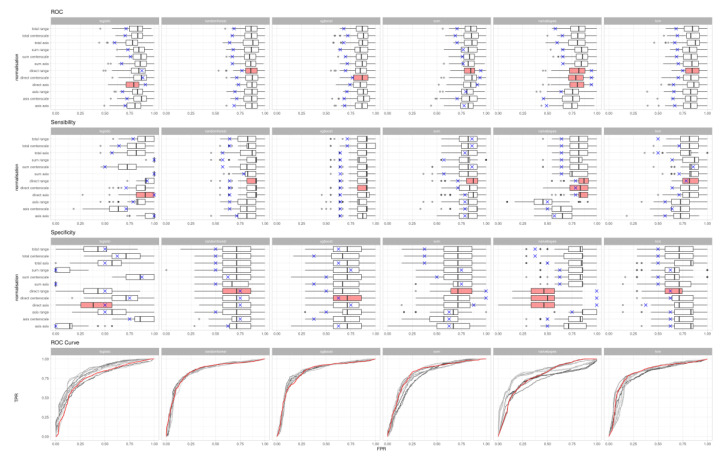
Comparison of area under curve (AUC), diagnostic sensitivity and specificity results on the training datasets for different normalisation processes. Boxplots and ROC curves refer to the training set. Blue cross shows the metric on the test set. Highlighted in red, the normalisation with the best ROC area under curve metric on the test set, for the given algorithm. Columns (algorithms) -> “logistic”: Logistic regression; “randomforest”: Random forest; “xgboost”: Extreme gradient boosting; “svm”: Support vector machines; “naivebayes”: Naïve bayes; “knn”: k-nearest neighbors rows (normalisations, also reported in Figure 1) -> “asis”: No normalisation applied; “sum”: Plasma bile acids (BAs) divided by the sum of the plasma BAs; “total”: Plasma BAs divided by the total bilirubin; “direct”: Plasma BAs divided by the direct bilirubin; “centerscale”: Mean-centering; “range”: 0–1 range.

The results of the different combinations of normalisation processes and algorithms on the training and test datasets are graphically displayed in Figure 2.

Table 1 shows results on the holdout set of the best-performing normalisation method of each model. Overall, the AUC, diagnostic sensitivity and specificity of the six models ranged between 0.75–0.95, 0.64–1.00 and 0.50–1.00, respectively.

**Figure 3:**
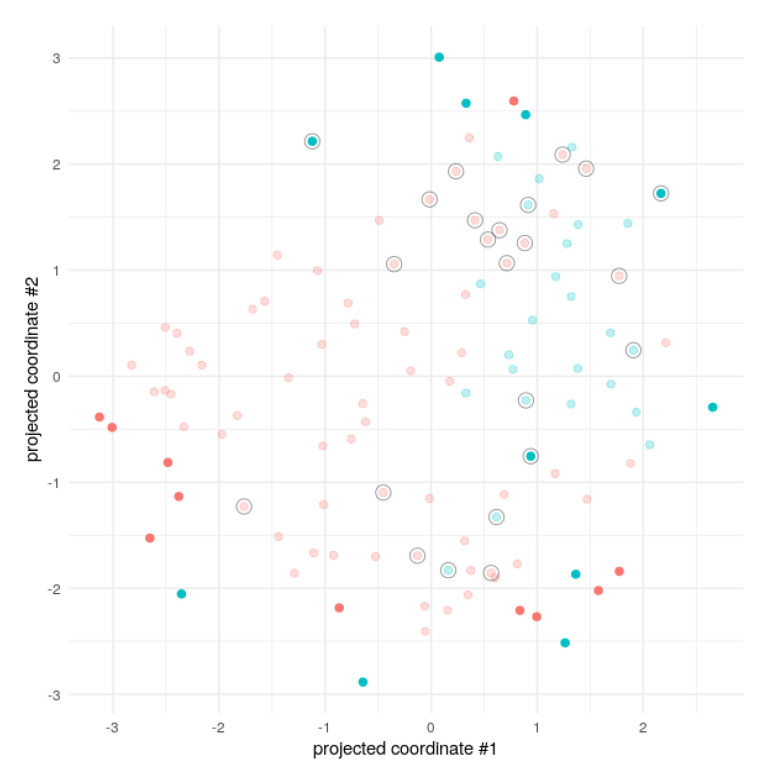
Dataset visual inspection using UMAP 2D projection of the best model (naïve bayes) for the best normalisation (plasma BAs divided by direct bilirubin, 0–1 range). Red dots: Patients with cholangiocarcinoma (CCA); blue dots: Patients with benign biliary diseases. Shadow points are the training set, highlighted points are the test/holdout set. Circled points are the misclassified ones.

The UMAP visualisation for the model characterised by best accuracy (i.e., AUC value) and diagnostic sensitivity for the best normalisation (plasma BAs divided by direct bilirubin, 0–1 range) was the Naïve bayes (Figure 3). This model, which was also characterised by 1.00 positive predictive value and 0.73 negative predictive value, displayed a globally excellent accuracy, as high as 86.4%.

## 4. Discussion

Although diagnostic and decision processes are still considered empirical and mostly based on physician’s reasoning in several clinical settings [1], AI and ML are increasing their influence in the industrial field as interesting sources of previously unknown information, for predicting users behaviours or increasing revenues and improving results [27]. Notably, a growing number of articles published in Medline-indexed journals are now using ML methods to perform complex operations, predictions and for identifying new information in healthcare settings [1]. In keeping with this new trend, we designed this study to investigate whether this approach could be potentially applied to the analysis of plasma BAs for discriminating benign from malignant biliary diseases [14].

The differential diagnosis of biliary strictures remains a challenge, since the currently available imaging procedures and cytologic examinations display almost limited sensitivity [28], so that a pre-operative assessment of malignancy would be highly advisable. In this perspective, development and implementation of innovative diagnostic tools, including the algorithm that we have developed in this study, may represent a step forward for a more appropriate patient management, by helping to plan the most effective treatments, also encompassing the need for and the type of surgery.

With the purpose of evaluating ML, we prioritised the results of the AUC and diagnostic sensitivity, since our aim was to generate a screening algorithm instead of focusing on specificity, which is more suitable for formulating a specific diagnosis of malignancy, which will remain instead for long within the histopathology domain [29]. Moreover, without a precise knowledge a priori on data distribution and how the different variables could influence the learning models, we decided to try different normalisation procedures (Figure 1), choosing only those performing better for comparison on holdout set (Table 1).

The naïve bayes was the ML model characterised by better diagnostic performance on the holdout set, displaying 0.95 AUC, 0.79 sensitivity, 1.00 specificity and 86.4% overall accuracy. Results were similar to RBF support vector machines (which had the same ROC metric, but lower diagnostic sensitivity and overall accuracy). Surprisingly, tree-based methods (such as random forest and extreme gradient boosting) showed a much lower accuracy, despite being considered within the top performing algorithms for complex datasets.

As concerns data normalisation, we found a general agreement among the different models using direct bilirubin concentration. This finding is not surprising given that post hepatic jaundice represents the most common biochemical abnormality in both benign and malignant biliary diseases. We further inspected the resulting models, both using UMAP Projection to visualise the dataset and the prediction (Figure 3), where no clear boundary between classes can be found, and checking the top model feature importance (where direct bilirubin, TLCA and HDCA are among the most important variables, whereas sex and TUDCA are of less influence).

For exhaustiveness, we also performed the same experiment on a reduced dataset, where the features have been selected according to random forest recursive feature elimination (rf-RFE) [30], achieving similar results in terms of best models and normalisation, but recording a 1–5% decay of diagnostic accuracy (data not shown).

One of the limitations of this study is that we decided to test our data only using some of the available ML models, more specifically using the most used and widespread. It is otherwise possible to test other ML algorithms or use ensemble methods (i.e., using at same time multiple models to obtain better results) [31]. The other important limitation of this study is the limited number of patients involved (90 patients during the training phase and 22 in the test set), so that additional studies on larger patient cohorts would be necessary to validate our preliminary findings.

In conclusion, the results of our study support the hypothesis that the analysis of BAs profile based on ML algorithms provides a promising contribution for characterisation of bile ducts diseases. Nevertheless, additional evidence is needed, encompassing also larger patient cohorts, before these algorithms can be used as routine tools for screening of patients at higher risk of malignant diseases.

## Figures and Tables

**Table 1:** Confusion matrices of the holdout set (*n* = 22) for the best normalisation process of each model.

Algorithm	Naïve Bayes	RBF Support Vector Machines	Logistic Regression	Random Forest	Extreme Gradient Boosting
**First normalisation**	Plasma BAs divided by the direct bilirubin	Plasma BAs divided by the direct bilirubin	Plasma BAs divided by the direct bilirubin	Plasma BAs divided by the direct bilirubin	Plasma BAs divided by the direct bilirubin
**Second normalisation**	0–1 range	0–1 range	No normalisation applied	0–1 range	Mean-centring
**True positives**	11	10	14	9	9
**True negatives**	8	8	4	6	5
**False positives**	0	0	4	2	3
**False Negatives**	3	4	0	5	5
**ROC area under curve**	0.95	0.95	0.91	0.77	0.77
**Accuracy**	0.864	0.818	0.818	0.682	0.700
**Sensitivity**	0.79	0.71	1.00	0.64	0.64
**Specificity**	1.00	1.00	0.50	0.75	0.63

## Abbreviations

BA (BAs)	Bile acid (Bile acids)
CCA	Cholangiocarcinoma
ML	Machine learning
AI	Artificial intelligence
UHPLC	Ultra (high) performance liquid chromatography
MS/MS	Tandem mass spectrometry
AUC	Area under curve
UMAP	Uniform manifold approximation and projection
ROC	Receiver operating characteristic
